# Supplementary material for: A genetically encoded probe for imaging nascent and mature HA-tagged proteins in vivo
Source: Nat Commun. 2019 Jul 3;10:2947. doi: 10.1038/s41467-019-10846-1 (PMC6610143; doi:10.1038/s41467-019-10846-1)
Supplement: Supplementary file 1 — Supplementary Information [file 41467_2019_10846_MOESM1_ESM.pdf]

Supplementary Information

A genetically encoded probe for imaging nascent and mature HA-tagged proteins *in vivo*

Zhao et al.

Supplementary Table 1

Primer sequences

Primer name	Sequence (5’-3’)
NZ-098	CGTCAGATCCGCTAGCGCTACCGGTCGCCACCATGTACCCTTATGATGTG
NZ-099	ACTGAACCTCCTCCACCTGCGGCCGCAGCGTAGTCCGGGACATCGTAC
NZ-100	CTGGGCCTCCGGAGCCTCCAGATCTAGCGTAGTCCGGGACATCGTAC
NZ-073	TGGACGAGCTGTACAAGTCCGGACTCAGATCTGGAGGCTCCGGAGGCGATGATGATATCGCCGC GCTCGTCGTCGACAACG
NZ-074	ATGATCAGTTATCTAGATCCGGTGGATCCCTTAGAAGCATTTGCGGTGGACGATGGAGGGGCCGG ACT
NZ-105	CGTCAGATCCGCTAGCGCTAC
NZ-106	CTTAGACACCATGGTGGCGACCGGTGATCCACCGCCTCCAGCGTA
Kv2.1-1	CTGCAGGCGATCGCCACGAAGCATGGCTCGCGC
Kv2.1-2	CGGGAGCACTAGGGATCAGAGTATCGTTTAAACGCTAGCT
NZ-092	CGTCAGATCCGCTAGCGCTACCGGTCGCCACCATGCCAGAGCCAGCGAAGTCTGCTC
NZ-093	ACTGAACCTCCTCCACCTGCGGCCGCCTTAGCGCTGGTGTACTTGGTGATG
NZ-094	CTGTACAAGTCCGGACTCAGATCTGGAGGCTCCGGAGGCTACCCGTACGATGTCCCGGAC
NZ-095	ATGATCAGTTATCTAGATCCGGTGGATCCCTTAAGCGTAGTCCGGGACATCGTACGGGTA
NZ-096	GCTGTACAAGTCCGGACTCAGATCTGGAGGCTCCGGAGGCTACCCTTATGATGTGCCCGATTATG C
NZ-097	GATCAGTTATCTAGATCCGGTGGATCCCTTAAGCGTAGTCCGGGACATCGTAC
NZ-075	TTTGTTTAACTTTAAGAAGGAGATATACATATGGCCGAGGTGAAGCTGGTGGAG
NZ-077	TGGTGGTGCTCGAGTGCGGCCGCCTTGTACAGCTCGTCCATGCCGAGAG
HAout-1	CCTCCGCCTCCACCAGCGTAATCTGAACTAGCGGTTCTGCCGCTGCTCACGGTCACCAGGGTGC CC
HAout-2	GGGCACCCTGGTGACCGTGAGCAGCGGCAGAACCGCTAGTTCAGATTACGCTGGTGGAGGCGG AGG

Supplementary Figures

χ^{HA}_{15F11} MAEVKLVESGGGLVKPGGSLKLSCAASGFTFSSYGMSWVRQTPEKRLEWVATISRGGSYT 60
χ^{HA}_{2E2} MAEVQLVESGGDLVKPGGSLKLSCAASGFTFSSYGMSWVRQTPDKRLEWVATISRGGSYT 60

χ^{HA}_{15F11} YYPDSVKGRFTISRDNAKNTLYLQMSSLRSEDTAIYYCARRETYDEKGFAYWGQGTTLTV 120
χ^{HA}_{2E2} YYPDSVKGRFTISRDNAKNTLYLQMSSLKSEDTAMYYCARRETYDEKGFAYWGQGTSVTV 120

χ^{HA}_{15F11} SSGGGGSGGGGSGGGGSDIVLTQSPASLTVSLGQRATISCKSSQSLLNSGNQKNYLTWYQ 180
χ^{HA}_{2E2} SSGGGGSGGGGSGGGGSDIVLTQSPASLAVSLGQRATISCKSSQSLLNSGNQKNYLTWYQ 180

χ^{HA}_{15F11} QKPGQPPKLLIYWASTRESGIPARFSGSGSGTDFTLNIHPVEEEDAATYYCQNDNSHPLT 240
χ^{HA}_{2E2} QKPGQPPKLLIYWASTRESGIPARFSGSGSGTDFTLNIHPVEEEDAATYYCQNDNSHPLT 240

χ^{HA}_{15F11} FGAGTKLEI 249
χ^{HA}_{2E2} FGGGTKLEI 249

Supplementary Figure 1 The sequence of frankenbodies. Six CDRs are highlighted in yellow. Mismatched amino acids are highlighted in green.

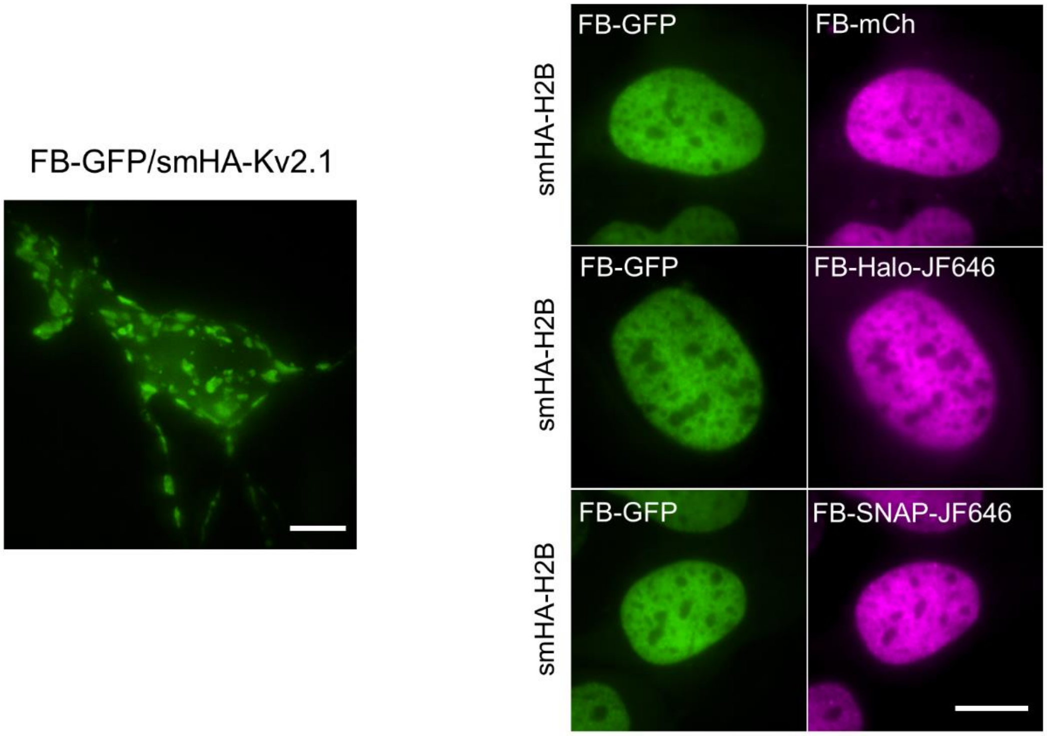

Supplementary Figure 2 Representative cell images of FB-GFP binding to HA-tagged proteins. Left: FB-GFP labels smHA-Kv2.1 (n=2 cells in one independent experiment); Right: Co-expression of FB-GFP (green) with other FB fused to different fluorescent proteins. From top to bottom: FB-GFP+FB-mCh (n=20), FB-GFP+FB-Halo-JF646 (n=20), FB-GFP+FB-SNAP-JF646 (n=20) in one independent experiment. Scale bars, 10 µm. Source data are provided as a Source Data file.

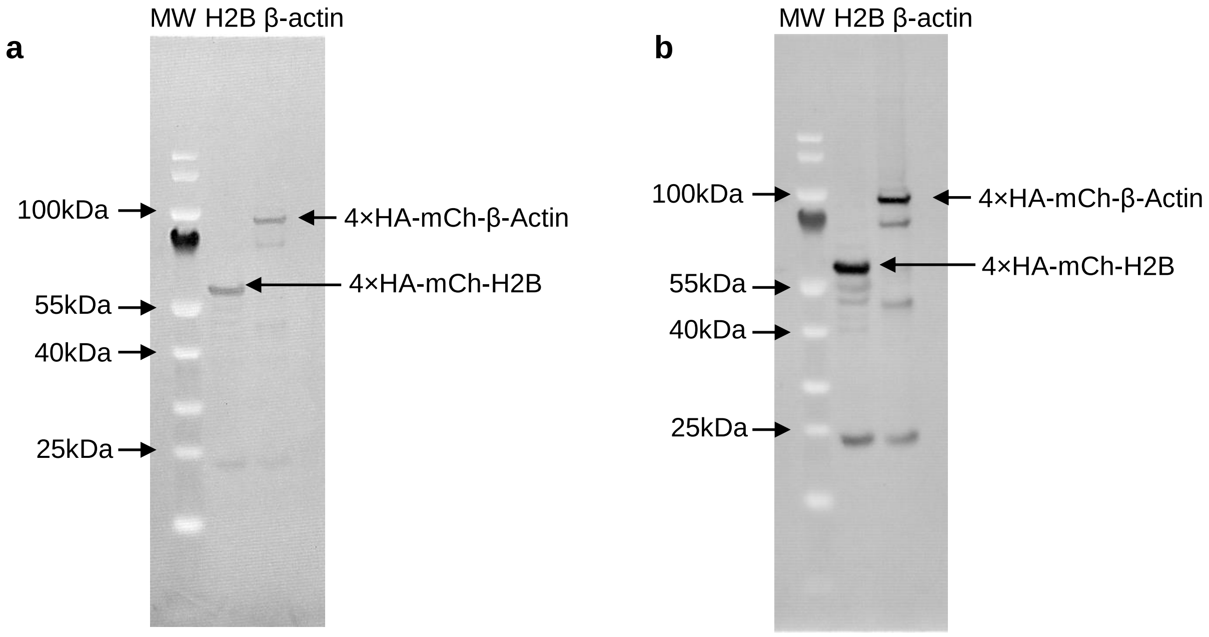

Supplementary Figure 3 Uncropped and unprocessed Western blots of HA-tagged H2B and β-actin. **a** Purified FB-GFP (1:2000 dilution, no secondary antibody) detected directly using GFP fluorescence; **b** Parental anti-HA antibody 12CA5 (1:2000 dilution) detected with secondary anti-mouse antibody/Alexa488 (1:5000 dilution).

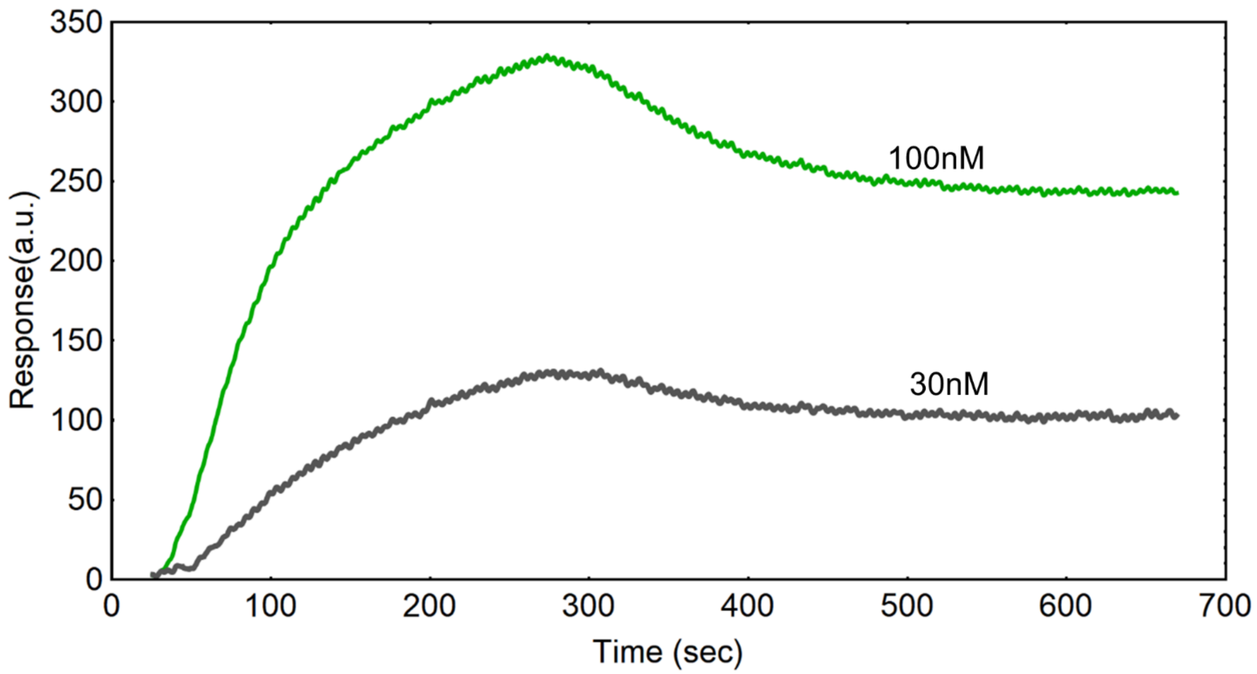

Supplementary Figure 4 Binding kinetics of frankenbody to HA tag *in vitro.* K_D =14.7±7.4 nM (Mean±SEM). Two independent experiments. Source data are provided as a Source Data file.

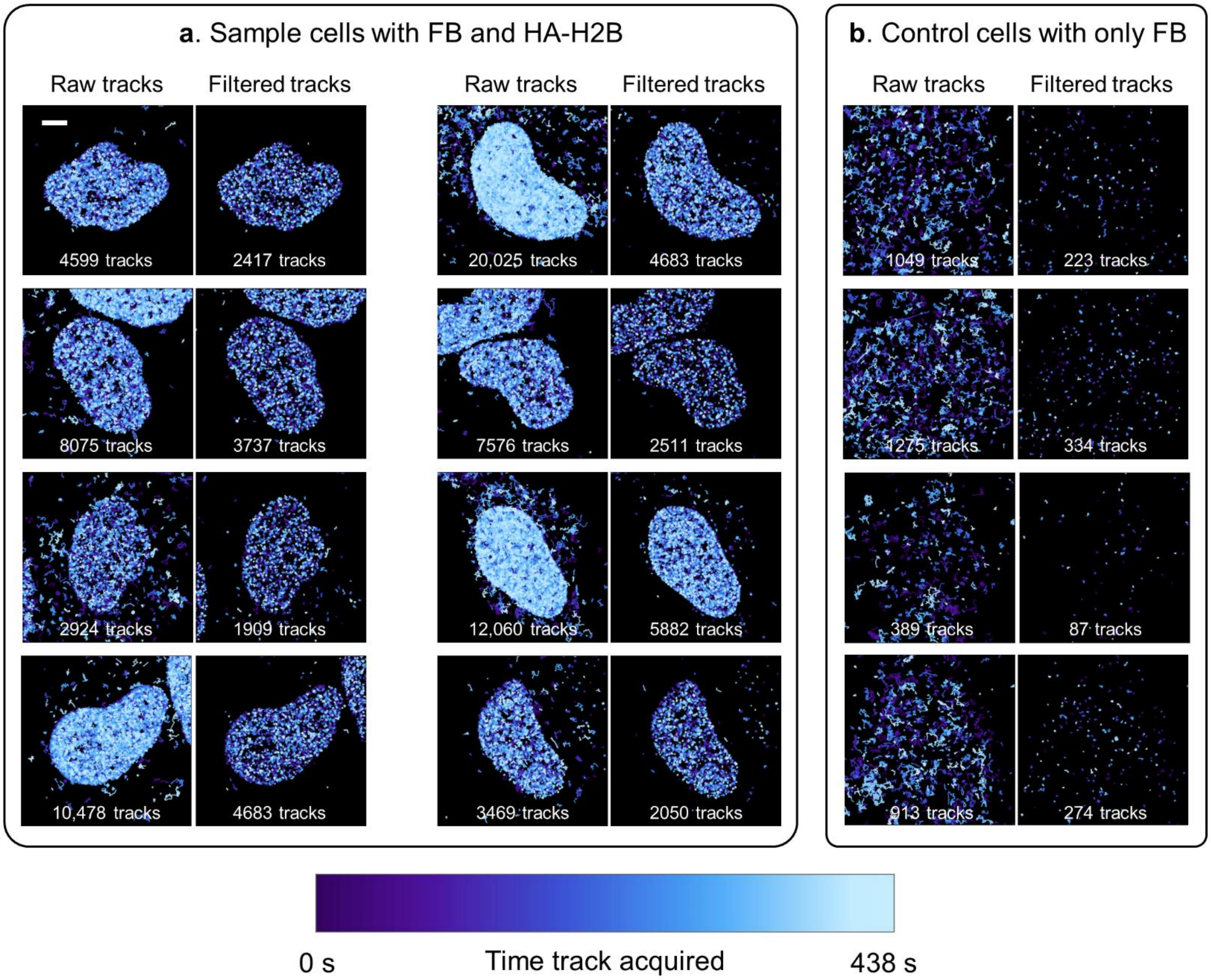

Supplementary Figure 5 Single molecule tracking of 1×HA-tagged proteins in living cells. **a** Image showing all single molecule tracks of Halo-tagged frankenbody (FB) in 3 independent experiments. To increase the density of tracks within cells, the TMR-Halo ligand was pretreated with 50mM sodium borohydride prior to staining. Tracks are color coded according to their time of acquisition (lighter is later during the movie). To ensure tracks represent FB bound to HA-H2B, a filter was used. The filter eliminated tracks of length less than 16 frames. Further, all jumps between frames had to be less than 220 nm. The mean track length is 38 ± 6 frames (mean ± SD). The number of tracks is shown at the bottom of each image. **b** The same in control cells loaded with FB but lacking HA epitopes in one independent experiment. Scale bar, 5 mm. Source data are provided as a Source Data file.

a

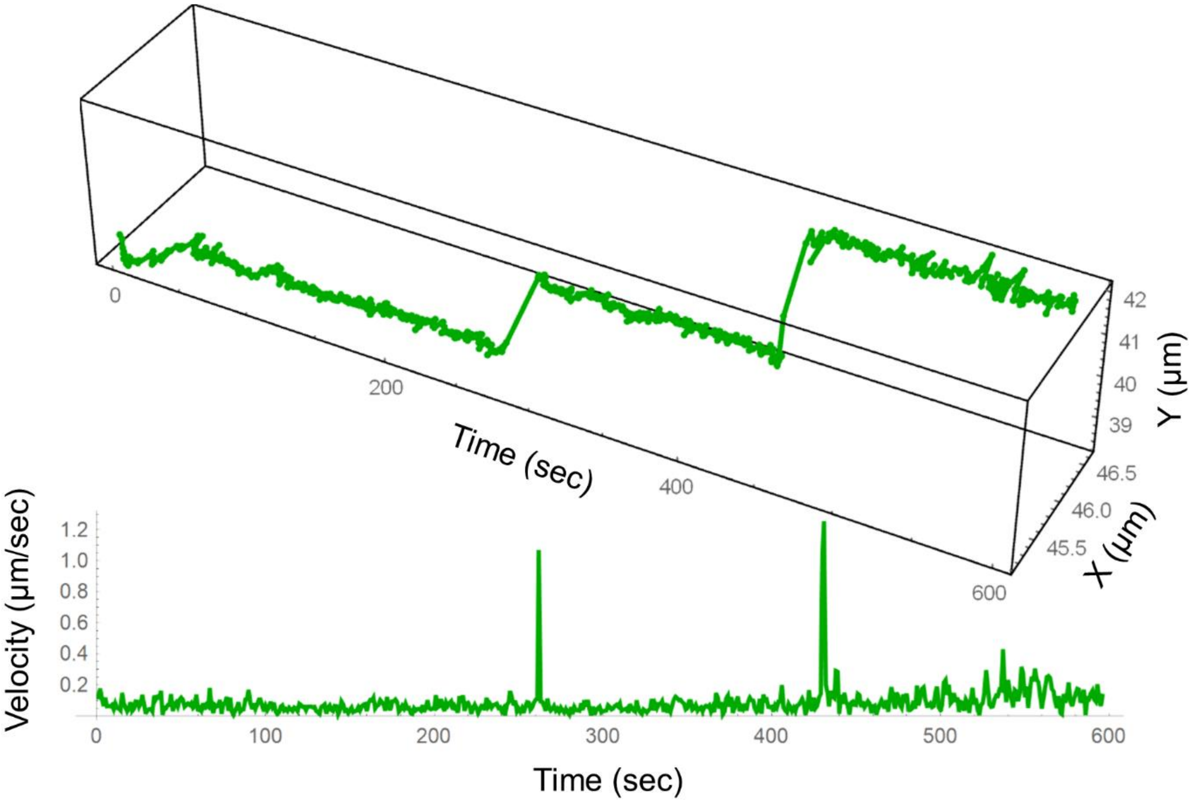

b

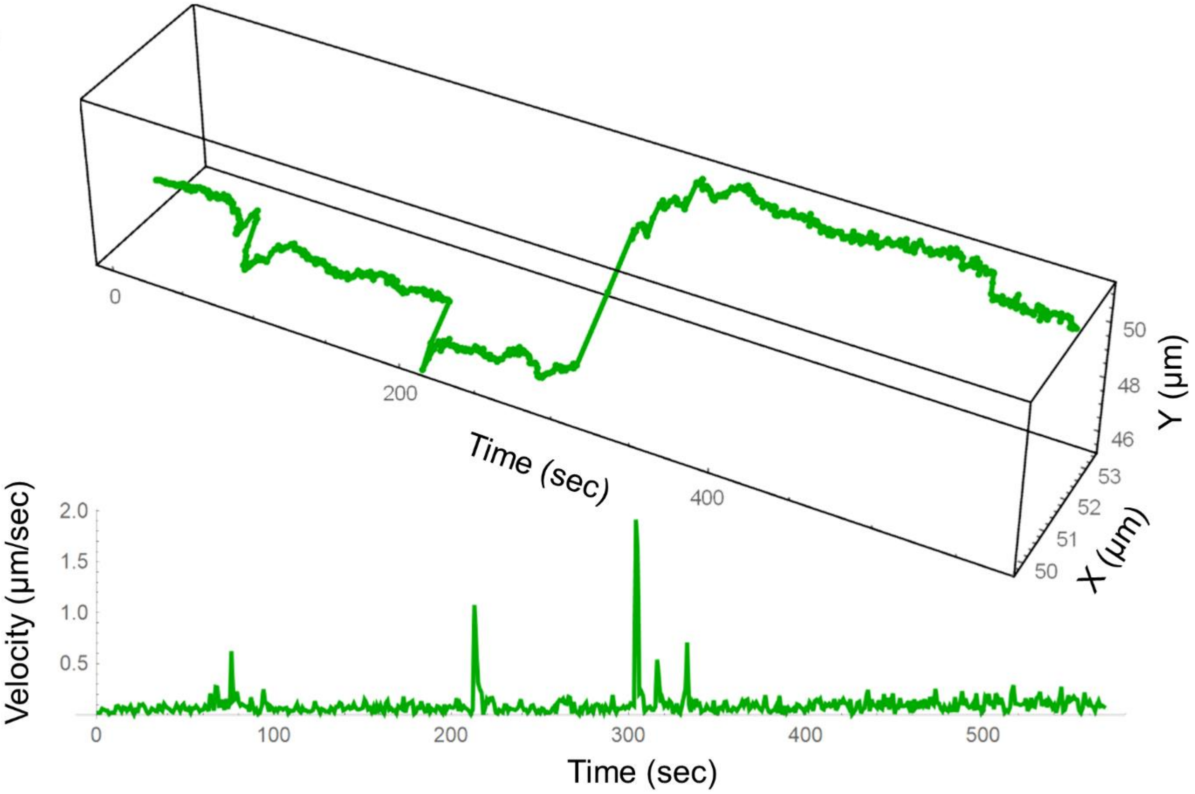

Supplementary Figure 6 Tracking motored translation spots with HA frankenbody in live neurons. **a** A representative translation spot in a neuron showing motored movement along neuron dendrites. Top: movement of the motored translation spot through time; bottom, velocity change through time. Sharp peaks indicate motored movement. **b** Another translation spot, as in **a**, but in a different neuron. Source data are provided as a Source Data file.

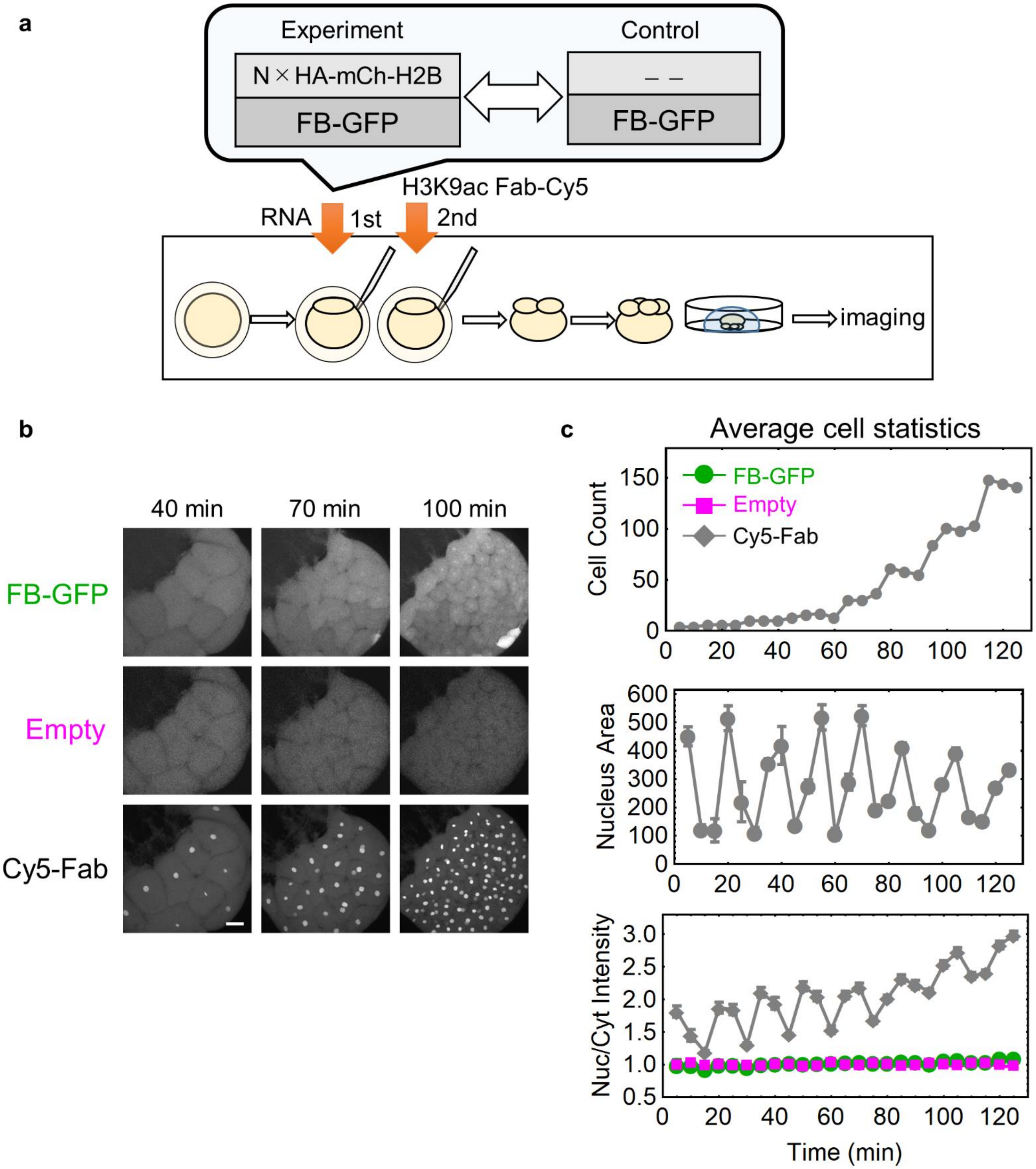

Supplementary Figure 7 HA frankenbody does not bind non-specifically in zebrafish embryos lacking HA epitopes. **a** A diagram depicting the timing of zebrafish embryo imaging experiments. Embryos were loaded with HA frankenbody (FB-GFP) and N×HA-mCh-H2B (N=1,4,10) (absent in control). **b** Sample max-projection images from a control zebrafish embryo with FB-GFP (green), but lacking target HA-mCh-H2B (Empty; magenta). Positive control Cy5-Fab marks histone acetylation in nuclei. **c** Cell count (top), average nuclear area (units of pixel^2 with one pixel = 662 nm, middle) and nuclear to cytoplasmic (Nuc/Cyt) ratio for all tracked nuclei. Supplementary Fig. 7 (left column) shows a repeat control. Error bars, SEM. Scale bar: 50 µm. Source data are provided as a Source Data file.

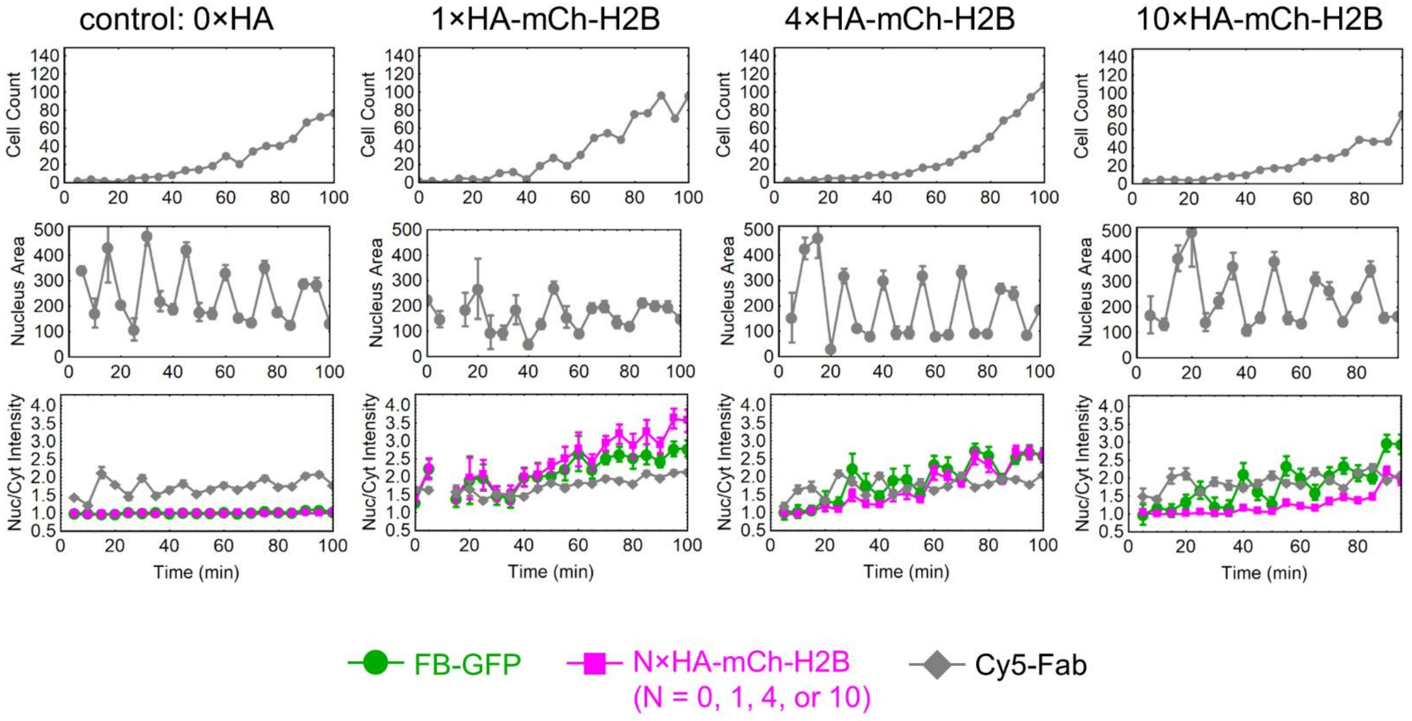

Supplementary Figure 8 FB-GFP signal improves with more HA epitopes present in Zebrafish embryos. Zebrafish embryos were injected with mRNA encoding frankenbody (FB-GFP) and N×HA-mCh-H2B (from left to right, N=0,1,4,10). Embryos were also injected with Cy5-Fab to mark histone acetylation in the nuclei. Cell count (top), average nuclear area (middle) and nuclear to cytoplasmic (Nuc/Cyt) ratio in all tracked cells through time. With N=1, the green FB-GFP curve nuclear to cytoplasm ratio is consistently lower than the magenta 1×HA-mCh-H2B. With N=4, the ratios are more equal. With N=10, the green nuclear to cytoplasm ratio is consistently higher than the magenta 10×HA-mCh-H2B. One zebrafish embryo in one independent experiment for each construct. Error bars, SEM between cells. Source data are provided as a Source Data file.
