# Supplementary material for: A genetically encoded probe for imaging nascent and mature HA-tagged proteins in vivo
Source: Nat Commun. 2019 Jul 3;10:2947. doi: 10.1038/s41467-019-10846-1 (PMC6610143; doi:10.1038/s41467-019-10846-1)
Supplement: Supplementary file 3 — Description of Additional Supplementary Files [file 41467_2019_10846_MOESM3_ESM.pdf]

Description of Additional Supplementary Files

File name: Supplementary Movie 1

Description: Related to Figure 2b right panel. 3D projection of the neuron co-expressing FB-GFP and 4×HA-mRuby-Kv2.1 in Fig. 2b, showing beautiful and clear peripheral membrane co-localization. Movie field of view is 65.29×62.98 μm.

File name: Supplementary Movie 2

Description: Related to Figure 5a. Single molecule tracking of 1×HA-tagged proteins in cells. H2B single molecules were labeled by FB-Halo-TMR (TMR was pretreated with $NaBH_4$). Images were acquired every 43.8 msec for 10,000 time points (movie duration is 45 sec, showing a 1,000 time points example). A 405 nm laser was pulsed every 10 sec, starting from 44 sec. Movie field of view is 33.28 × 33.28 μm.

File name: Supplementary Movie 3

Description: Related to Figure 6b. Tracking single mRNA translation in living U2OS cells. Nascent chain was labeled by FB-GFP shown in green. mRNA was labeled by MCP-Halo-JF646 shown in magenta. Images were acquired every 10 sec (movie duration is 9 min 50 sec). Movie field of view is 66.56 × 66.56 μm.

File name: Supplementary Movie 4

Description: Related to Figure 6c. Puromycin treatment of FB-GFP tracking single mRNA translation in living U2OS cells. Puromycin was added at 1 min 40 sec, right before frame 11. Images were acquired every 10 sec (movie duration is 4 min 50 sec). Movie field of view is 46.15 × 43.03 μm.

File name: Supplementary Movie 5

Description: Related to Figure 6e. Puromycin treatment of FB-Halo tracking single mRNA translation in living U2OS cells. Puromycin was added at 1 min 40 sec, right before frame 11. Images were acquired every 10 sec (movie duration is 9 min 50 sec). Movie field of view is 66.56 × 66.56 μm.

File name: Supplementary Movie 6

Description: Related to Figure 6f. Puromycin treatment of FB-mCh tracking single mRNA translation in living U2OS cells. Puromycin was added at 1 min 40 sec, right before frame 11. Images were acquired every 10 sec (movie duration is 9 min 50 sec). Movie field of view is 66.56 × 66.56 μm.

File name: Supplementary Movie 7

Description: Related to Figure 7b. Multiplexed imaging of single mRNA translation dynamics. 2 orthogonal pairs of probe/translation reporter: Sun-GFP/SunTag-Kif18b (green) and FB-mCh/smHA-KDM5B (magenta) track single mRNA translation simultaneously in one living U2OS cell. Images were acquired every 2 sec (movie duration is 3 min 18 sec). Movie field of view is 66.56 × 66.56 μm.

File name: Supplementary Movie 8

Description: Related to Figure 8b. Frankenbody tracks single mRNA translation in living neurons. A dendrite of a sample living neuron expressing frankenbody (FB-GFP) and the smHA-KDM5B translation reporter. Images were acquired every 14 sec (movie duration is 23 min 25 sec). Movie field of view is 66.56 × 66.56 μm.

File name: Supplementary Movie 9

Description: Related to Figure 8c. Puromycin treatment of FB-GFP tracking single mRNA translation in living neurons. Puromycin was added at 3 min 20 sec, right before frame 6. Image was acquired every 40 sec (movie duration is 14 min). Movie field of view is 33.8 × 33.67 μm.

File name: Supplementary Movie 10

Description: Related to Figure 9. HA frankenbody binds target epitopes in zebrafish embryos. Single-channel movies showing the HA frankenbody binding specifically to target HA-epitopes in zebrafish embryos (Left top: FB-GFP; Right top: 4×HA-mCh-H2B; Left bottom: Cy5-Fab). A merge of all 3 channels is also shown (Right bottom; FB-GFP: green; 4×HA-mCh-H2B: red; Cy5-Fab: blue). Images were acquired every 5 min (movie duration is 80 min). Movie field of view is 423.68 × 423.68 μm.

File name: Supplementary Movie 11

Description: Related to Supplementary Figure 6. HA frankenbody does not bind non-specifically in zebrafish embryos lacking HA epitopes. Single-channel movies showing the HA frankenbody in zebrafish embryos lacking HA-epitopes (Left top: FB-GFP; Right top: empty; Left bottom: Cy5-Fab). A merge of all 3 channels is also shown (Right bottom; FB-GFP: green; empty: red; Cy5-Fab: blue). Images were acquired every 5 min (movie duration is 80 min). Movie field of view is 423.68 × 423.68 μm.
