# Supplementary material for: A genetically encoded probe for imaging nascent and mature HA-tagged proteins in vivo
Source: Nat Commun. 2019 Jul 3;10:2947. doi: 10.1038/s41467-019-10846-1 (PMC6610143; doi:10.1038/s41467-019-10846-1)
Supplement: Supplementary file 15 — Reporting Summary [file 41467_2019_10846_MOESM15_ESM.pdf]

nature research

Corresponding author(s): Timothy J. Stasevich

Last updated by author(s): May 8, 2019

Reporting Summary

Nature Research wishes to improve the reproducibility of the work that we publish. This form provides structure for consistency and transparency in reporting. For further information on Nature Research policies, see Authors & Referees and the Editorial Policy Checklist.

Statistics

For all statistical analyses, confirm that the following items are present in the figure legend, table legend, main text, or Methods section.

n/a	Confirmed	
☐	☒	The exact sample size (*n*) for each experimental group/condition, given as a discrete number and unit of measurement
☐	☒	A statement on whether measurements were taken from distinct samples or whether the same sample was measured repeatedly
☐	☒	The statistical test(s) used AND whether they are one- or two-sided<br>*Only common tests should be described solely by name; describe more complex techniques in the Methods section.*
☒	☐	A description of all covariates tested
☒	☐	A description of any assumptions or corrections, such as tests of normality and adjustment for multiple comparisons
☐	☒	A full description of the statistical parameters including central tendency (e.g. means) or other basic estimates (e.g. regression coefficient) AND variation (e.g. standard deviation) or associated estimates of uncertainty (e.g. confidence intervals)
☐	☒	For null hypothesis testing, the test statistic (e.g. *F*, *t*, *r*) with confidence intervals, effect sizes, degrees of freedom and *P* value noted<br>*Give P values as exact values whenever suitable.*
☒	☐	For Bayesian analysis, information on the choice of priors and Markov chain Monte Carlo settings
☒	☐	For hierarchical and complex designs, identification of the appropriate level for tests and full reporting of outcomes
☐	☒	Estimates of effect sizes (e.g. Cohen's *d*, Pearson's *r*), indicating how they were calculated

Our web collection on statistics for biologists contains articles on many of the points above.

Software and code

Policy information about availability of computer code

Data collection	Raw microscopy images and movies are available for all Figures. Images were acquired using open-source Micro-Manager software (1.4.22) or the commercially available softwares SlideBook (6.0.17) and FLUOVIEW (4.2)
Data analysis	FRAP data were analyzed by easyFRAP-web version 1.3. The other data were analyzed by Fiji ImageJ and with custom Wolfram Mathematica code (version 11.1.1). All custom code is available upon request.

For manuscripts utilizing custom algorithms or software that are central to the research but not yet described in published literature, software must be made available to editors/reviewers. We strongly encourage code deposition in a community repository (e.g. GitHub). See the Nature Research guidelines for submitting code & software for further information.

Data

Policy information about availability of data

All manuscripts must include a data availability statement. This statement should provide the following information, where applicable:

- Accession codes, unique identifiers, or web links for publicly available datasets
- A list of figures that have associated raw data
- A description of any restrictions on data availability

The source data underlying Figures 1e, 2a, 2d, 2f, 4b, 4d, 5b-c, 6c, 6e-f, 7c, 8b-e, 9b-c and Supplementary Figures 4, 5, 6, 7c and 8 are provided as a Source Data file. The raw images were deposited to figshare and can be accessed at DOI: 10.6084/m9.figshare.8072438. All other data are included in the manuscript and/or supplemental materials are available from the corresponding authors upon reasonable request.

Field-specific reporting

Please select the one below that is the best fit for your research. If you are not sure, read the appropriate sections before making your selection.

☒ Life sciences ☐ Behavioural & social sciences ☐ Ecological, evolutionary & environmental sciences

For a reference copy of the document with all sections, see nature.com/documents/nr-reporting-summary-flat.pdf

Life sciences study design

All studies must disclose on these points even when the disclosure is negative.

Sample size	Sample sizes are different for each figure and the detailed information is listed in figure legends.
Data exclusions	We excluded cells that were not transfected with our frankenbody or reporter constructs, contained too many translation spots to track, or contained too much mature nuclear protein to visualize translation spots.
Replication	The replication information is listed in figure legends.
Randomization	No randomization was performed in this study
Blinding	No blinding was done in this study.

Reporting for specific materials, systems and methods

We require information from authors about some types of materials, experimental systems and methods used in many studies. Here, indicate whether each material, system or method listed is relevant to your study. If you are not sure if a list item applies to your research, read the appropriate section before selecting a response.

Materials & experimental systems

n/a	Involved in the study	
☐	☒	Antibodies
☐	☒	Eukaryotic cell lines
☒	☐	Palaeontology
☐	☒	Animals and other organisms
☒	☐	Human research participants
☒	☐	Clinical data

Methods

n/a	Involved in the study	
☒	☐	ChIP-seq
☒	☐	Flow cytometry
☒	☐	MRI-based neuroimaging

Antibodies

Antibodies used	anti-HA antibody 12CA5. We purchased it from Sigma-Aldrich with REF number: 11583816001 and lot number: 25621800
Validation	The vendor should validate the antibody. We didn't do any antibody validation.

Eukaryotic cell lines

Policy information about cell lines

Cell line source(s)	U-2 OS (ATCC® HTB-96™)
Authentication	the commercial available cell line was authenticated by the cell line provider.
Mycoplasma contamination	The cell lines were not tested for mycoplasma contamination.
Commonly misidentified lines (See ICLAC register)	No commonly misidentified lines were used in this study.

Animals and other organisms

Policy information about studies involving animals; ARRIVE guidelines recommended for reporting animal research

Laboratory animals	Zebrafish (wild-type of AB strain)

Wild animals	The study did not involve wild animals.
Field-collected samples	The study did not involve samples collected from field.
Ethics oversight	All zebrafish experiments have been approved by the Tokyo Tech Genetic Experiment Safety Committee (I2018001) and animal handling is operated according to the guidelines.

Note that full information on the approval of the study protocol must also be provided in the manuscript.
